# Current Applications of Artificial Intelligence in Cleft Care: A Scoping Review

**DOI:** 10.3389/fmed.2021.676490

**Published:** 2021-07-28

**Authors:** Harnoor Dhillon, Prabhat Kumar Chaudhari, Kunaal Dhingra, Rong-Fu Kuo, Ramandeep Kaur Sokhi, Mohammad Khursheed Alam, Shandar Ahmad

**Affiliations:** ^1^Centre for Dental Education and Research, All India Institute of Medical Sciences, New Delhi, India; ^2^Medical Device Innovation Centre, National Cheng Kung University, Tainan, Taiwan; ^3^College of Dentistry, Jouf University, Sakakah, Saudi Arabia; ^4^School of Computational and Integrative Sciences, Jawaharlal Nehru University, New Delhi, India

**Keywords:** artificial intelligence, cleft lip, cleft palate, craniofacial anomalies, machine learning, neural network

## Abstract

**Objective:** This scoping review aims to identify the various areas and current status of the application of artificial intelligence (AI) for aiding individuals with cleft lip and/or palate.

**Introduction:** Cleft lip and/or palate contributes significantly toward the global burden on the healthcare system. Artificial intelligence is a technology that can help individuals with cleft lip and/or palate, especially those in areas with limited access to receive adequate care.

**Inclusion Criteria:** Studies that used artificial intelligence to aid the diagnosis, treatment, or its planning in individuals with cleft lip and/or palate were included.

**Methodology:** A search of the Pubmed, Embase, and IEEE Xplore databases was conducted using search terms artificial intelligence and cleft lip and/or palate. Gray literature was searched using Google Scholar. The study was conducted according to the PRISMA- ScR guidelines.

**Results:** The initial search identified 458 results, which were screened based on title and abstracts. After the screening, removal of duplicates, and a full-text reading of selected articles, 26 publications were included. They explored the use of AI in cleft lip and/or palate to aid in decisions regarding diagnosis, treatment, especially speech therapy, and prediction.

**Conclusion:** There is active interest and immense potential for the use of artificial intelligence in cleft lip and/or palate. Most studies currently focus on speech in cleft palate. Multi-center studies that include different populations, with collaboration amongst academicians and researchers, can further develop the technology.

## Introduction

Artificial intelligence (AI) is an application of computers to perform tasks that require human intelligence and discernment. Systems that use AI are built on mathematical models applied to the given data to recognize patterns and make decisions regarding the data, such as classifying it into a given category. Artificial intelligence systems physically consist of networks of processors meant to emulate biological systems (1). One of the earliest applications of AI in medicine was the development of the MYCIN system. It was called so as the names of most antibiotics at the time had the suffix -mycin. This was an expert system, a type of AI, designed to diagnose blood infections using more than 450 rules (2). Such systems are static and need manual updates, or they become obsolete over time. Recently, with advances in processing power and the availability of big data, the focus has shifted to obtaining large amounts of unprocessed information and using artificial intelligence to extract useful information that is otherwise not obvious (3).

In medicine, increasing digitization of medical data and documentation combined with the ease of managing digital record keeping using systems such as eClinicalWorks PM (USA), ECLIPSE Practice Management Software (MPN Software Systems, USA), has resulted in the generation of large volumes of data which can be processed using AI to discover previously unknown or, provide insights on, clinically relevant information. This will help improve decision-making in healthcare. The tasks for which this technology has been applied are of a preliminary nature (4). It has been used in medicine to aid diagnosis, interpretation of medical images, treatment planning, and prediction of treatment outcomes. Research in the area appears to be dispersed over various fields (5).

According to the National Institute of Health, cleft lip and/or palate (CL/P) is one of the most common birth defects that can occur as an isolated condition or as a part of a syndrome (6). The developing orofacial region is susceptible to abnormal development, with CL/P being the second most common birth defect in general (7). The incidence of cleft lip and palate is believed to be around 1 in 600 to 800 births (8–10). It is estimated that nearly 7.3 million people worldwide have orofacial clefts, and almost 220,000 cases are added every year (7). In low and middle-income countries, the incidence is much higher than in developed countries (11). According to the World Health Organization, cleft lip and palate is a major oral health condition contributing to the global burden of oral disease, with complete rehabilitation possible if treated appropriately (7, 12). The occurrence of a cleft does not result in an anatomic problem alone. Secondary issues such as speech and hearing problems, increased susceptibility to dental caries, difficulty eating—with associated malnutrition, and orthodontic problems also occur (13, 14). The handicaps associated with CL/P are medical and social. It has been found that different cultures, especially in developing countries, attribute the cause of the CL/P to superstition. This contrasts with countries where healthcare is more developed, and the reason is known to be scientific (15). In the long term, individuals with CL/P have adversely affected quality of life and long-term health. The need for additional healthcare and costs add to this burden (15, 16). At every stage of treatment, these individuals require constant monitoring with coordination between the cleft care team, the child, and parents (17). The caregivers are not immune to the emotional upheaval affected by the condition either. A qualitative study that explored the parents' experience of having a child with cleft lip and palate found that they had mixed feelings of happiness with despair and guilt, such as “*My first reaction was shock. I wasn't really prepared. We didn't think we'd have a child with a cleft palate. It was a funny feeling. I thought he was ugly but sweet at the same time*.” This study highlights the need for education and emotional support to help parents adapt to a child born with a deformity (18).

Modern technology has immense potential to help individuals with cleft lip and/or palate. It can help dissipate information, provide support to individuals and their caregivers, and remove communication barriers between specialists and individuals needing care. It can also aid doctors in areas with scant resources in the form of equipment or workforce to provide necessary care to individuals. Recent literature describes the use of digital technologies, such as using digital nasoalveolar molding (NAM) therapy (which consists of a series of plates used to reduce the severity of the nasolabial defect) will allow standard care while lowering the rate of infection, the need for specialist consultations, and the use of equipment (19). Case reports and series have described the usage of digital workflows, which can ease the burden of cleft on infants and their parents (20, 21).

It is a well-known fact that health care systems are not uniformly developed throughout the world (22). Accessibility to the internet, on the other hand, is available in most places. This calls for an amalgamation of medical knowledge with modern technology to reduce the burden of treatment on the affected individuals and their caregivers. While AI cannot replace an experienced and qualified medical professional, it can play a huge role in providing adequate resources to healthcare workers who are better positioned to help people in remote areas. It can make treatment more accessible, predictable and affordable for everyone.

A scoping review is a structured review of the available literature to provide an overview or map the research available, indicate the volume of literature available, and identify focus areas (23). The use of AI in cleft care is seeing a growing interest from the research community with the increasing applications of AI in dental and medical diagnosis, genomic predictions of diseases, and treatment needs and outcomes. This review aimed to identify the current research areas in the use of AI in cleft care and to recognize areas with a possibility of using AI in the future. Therefore, we conducted a scoping review to identify the current applications of AI for individuals with cleft lip and/or palate (CL/P).

## Methodology of the Review

This review was conducted following the Joana Briggs Institute guidelines for a scoping review (24). The reporting of results is as per the PRISMA statement extension for Scoping Reviews (25).

### Inclusion Criteria

The studies included in this review were selected according to the participants, concept, and context (PCC) criteria (24). The studies that aimed to benefit participants with CL/P by applying AI at any stage of the condition were included. Thus, the criteria were:

*Participants*—Individuals with isolated or syndromic cases of cleft lip and/or palate, or data derived from these participants.
*Concept*—Use of artificial intelligence for performing/augmenting/testing a procedure or diagnosis or assessment/prediction of treatment outcome/need.
*Context*—Use of artificial intelligence at any stage for individuals of cleft lip and/or palate.
*Types of Studies*—Literature that would be included consists of conference papers, experimental studies, quasi-experimental studies, exploratory studies, randomized/non-randomized controlled trials, cross-sectional and qualitative studies.

### Review Question

The question addressed by this review was, “*What are the current applications of artificial intelligence for individuals with cleft lip and/or palate?”*

### Search Strategy

A search of the following databases was done: MEDLINE (via OVID), Embase, PubMed, Cochrane Central Register of Clinical Trials (CENTRAL), and Cochrane Database of Systematic Reviews (CDSR) on 29/11/2020. Gray literature was searched using Google Scholar. The IEEE (Institute of Electrical and Electronics Engineers) database, IEEE Xplore, was also searched on 01/01/2021. Besides, a hand search of the selected studies was also carried out to identify additional publications by cross-referencing. The keywords used in the search were: (artificial intelligence OR machine learning) AND (cleft lip OR cleft palate). No time restrictions were applied to any database searched. Articles written in the English language were included.

Search results were screened based on their titles and abstracts to identify potentially relevant studies, and duplicates were removed using EndNote (Clarivate Analytics, Australia). The studies were then screened based on their abstracts, and those found to be relevant to the search question were examined based on the inclusion criteria.

## Results

**Figure 1:**
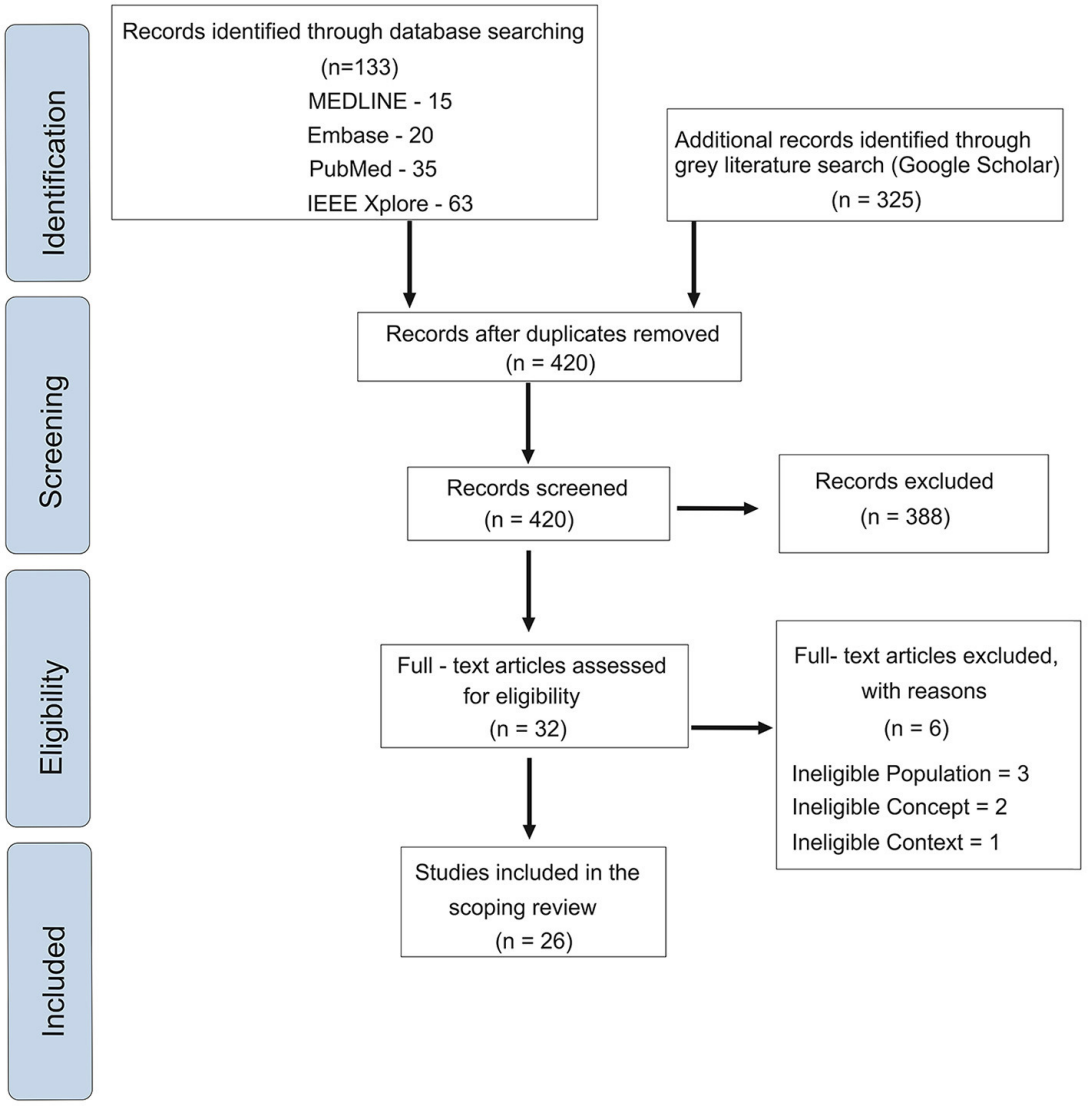
Workflow of the scoping review.

An initial search of the databases and the gray literature provided 458 articles. These were screened based on their titles and abstracts. After screening and removal of duplicates, 32 articles were evaluated by full-text reading. The PRISMA workflow diagram highlighting each step is provided in Figure 1. After applying the eligibility criteria, 26 articles were included. Based on the objective of the research and applications of AI in the various aspects of the care for individuals with cleft lip and/or palate, we categorized the included studies into five categories, namely (i) Risk of Development, (ii) Diagnosis, (iii) Pre-surgical Orthopedics, (iv) Speech assessment, and (v) Surgery.

All studies were performed at single centers with data from the local population. The highest number of studies were in the area of speech assessment in individuals with cleft palate. Maximum studies were done in China, with studies from the country in each of the areas identified. The characteristics of the studies in all these five categories are presented in the following sections and in Table 1.

**Table 1:** Characteristics of Included studies.

**S.No**	**References**	**Country**	**Aim**	**Age group**	**Sample size**	**Mode of AI used**	**Inputs employed**	**Method**	**Outcome**
**I. RISK OF DEVELOPING CLEFT LIP AND/OR PALATE**									
1.	Shafi et al. (26)	Pakistan	Predict the risk of development of cleft lip and palate (CLP)	N/A	1,000 (500 in each group)	Multilayer perceptron (MLP), k nearest neighbor (kNN), decision tree and random forest (RF)	Answers to questionnaires	A questionnaire was used to elicit data on 36 input features from mothers, half of whom had cleft babies while half were controls. Data was prepared and different predictive models were applied. Accuracy of the results obtained with each were evaluated.	MLP model with three hidden layers and 28 perceptrons in each gave the highest accuracy of classification (92.6%) on test data.
2.	Machado et al. (27)	Brazil	Predict the genetic risk of development of non-syndromic cleft lip with or without palate (NSCL ± P)	N/A	722 participants with NSCL ± P and 866 without NSCL ± P	RF and Neural Networks (NN)	Single Nucleotide Polymorphisms (SNP)	A model for genetic risk assessment of NSCL ± P in the Brazilian population by subjecting 72 known SNPs to RF which was used to determine significant SNPs. A NN was used to confirm the predictive model. Interactions among the SNPs were evaluated using multiple regression.	13 SNPs were highly predictive to identify individuals with NSCL ± P in the Brazilian population after RF and NN analysis. The combination of these was able to separate NSCL ± P subjects from controls with an accuracy of 94.5%. SNP–SNP interaction showed 13 significant pairs.
3.	Liu et al. (28)	China/USA	Assess gene interactions leading to development of NSCL ± P	N/A	806 patient–parent trios	Logic regression	SNPs	173 SNPs in and around eight genes were analyzed. A genotype to genotype two way and multi way interaction of SNPs from different genes involved in cell adhesion was analyzed using a machine learning algorithm.	Two way and multiway interactions between three genes–ACTN1, CTNNB1 and CDH1, contributed toward risk of NSCL ± P.
4.	Baker et al. (29)	U.S.A	Understand the mechanism of development of cleft palate via toxicology	N/A	500 chemicals	Data mining	High thoroughput screening data and chemical structural descriptors	A database of cleft active and inactive chemicals with identifiers such as gene scores and chemotypes was made. This was used to model a dataset for machine learning. A data mining software was used to extract indicators suggesting molecular initiating events leading to the adverse outcome of cleft palate.	Six molecular initiating events, each associated with a cluster of chemicals, were identified which could lead to an adverse outcome of cleft palate. The pathways for the same were also identified.
5.	Zhang et al. (30)	China	Assessment of genetic risk of developing NSCL ± P using SNPs	Infants	587 (Han and Uygur populations)	Support Vector Machines (SVM), logistic regression (LR), naïve bayes, decision trees, RF, k-NN, artificial neural network (ANN)	SNPs	43 SNP candidates were examined and their diagnostic ability in genetic risk assessment in the Chinese populations was validated using machine learning methods. From these, a panel of 24 SNPs was evaluated further after manual selection for risk assessment efficiency. This was done by sequential removal or addition of an SNP each time the LR based model was trained.	The LR model gave the best results for genetic risk assessment in the Han population while the Uyghur population obtained better results using the SVM. In the Uyghur population, the best results were obtained using the relative risk scoring model. Assessment efficiency showed that SNPs in three genes involved in folic acid and vitamin A synthesis play an important role in incidence of NSCL ± P.
6.	Li et al. (31)	USA	Identifying gene interactions that risk the development of NSCL ± P	N/A	895 (Asian) and 681 (European) patient–parent trios	RF and Logic regression	SNPs	RF was used to identify plausible SNPs in Asian and European populations. Potential genotype to genotype interactions were studied by machine learning and applied to NSCL ± P case parent trios focusing on the WNT pathway and those identified by genome wide association studies.	The study found evidence of interaction between SNPs in MAFB and WNT5B genes in both populations. WNT5B may also interact with markers in 8q24 region in Europeans and WNT5A may interact with markers in IRF6 and nearby open-reading frame C1orf107 in Asians.
**II.DIAGNOSIS OF CLEFT LIP AND/OR PALATE**									
7.	Jurek et al. (32)	Poland	Identify cleft palate in a prenatal ultrasound	Fetuses (11–13 weeks of gestation)	49 (36 non-cleft participants and 13 with CP)	Syntactic Pattern Recognition	Processed image of the fetal palate	Ultrasound images were used as input which were subject to processing for detecting the contour of the palate. Echogenicity histograms were generated by sequential image analysis. These were used to classify fetal palates as physiologic or pathologic.	The proposed method was able to identify 81.6% of the images effectively.
8.	Zhang et al. (33)	China	Estimate alveolar cleft defect voume prior to secondary alveolar bone grafting	11 ± 2.8 years	21 CBCTs	Deep Neural Network (DNN)	CBCT images	A partial non-rigid registration- based framework was used to determine the volume of bone missing in the alveolar cleft. The system was compared to other main stream non-rigid registration methods. The consistency between the estimate made by using the proposed system and ground truth was evaluated using the Dice similarity coefficient (DSC).	A completely automated system that can estimate bone volume in a cleft of the alveolus was created. The DSC of the proposed method was between 0.88 for the maxilla and 0.83 for the cleft. The relative volume error of the system (8%) was less than any of the mainstream systems.
9.	Alam and Alfawzan (34)	Saudi Arabia	Assess radiographic characteristics in participants with cleft lip and palate	13.29 ± 3.52 to 14.32 ± 4.46 years	123 (31 non-cleft and 92 participants with clefts)	AI based software (Webceph, Korea) was used	Lateral Cephalometric Radiographs	Radiographic characteristics of sella turcica of all the groups: unilateral CLP (UCLP), bilateral CLP (BCLP), unilateral cleft lip and alveolus (UCLA) and unilateral cleft lip (UCL), were compared and skeletal malocclusion was identified in subjects with bridging of sella turcica using an AI based software.	Sella turcica bridging, Skeletal class III malocclusions were more common in cleft subjects. Measurements of sella turcica were lowest in participants with bilateral cleft lip and palate followed by non-cleft individuals.
10.	Alam and Alfawzan (35)	Saudi Arabia	Assess dental characteristics in participants with cleft lip and palate	13.29 ± 3.52 to 14.32 ± 4.46 years	123 (31 non-cleft and 92 participants with cleft)	AI based software (Webceph, Korea) was used	Lateral Cephalometric Radiographs	An AI based software was used to assess dental characteristics among groups (non-cleft, BCLP, UCLP, UCLA, UCL). The results were statistically analyzed.	Of the 14 dental characteristics evaluated, eight were significantly altered in non-syndromic cleft individuals.
11.	Agarwal et al. (36)	U.S.A	Detect cleft lip on images	Newborns/children	1,451 images	Convolutional Neural Network (CNN) and SVM	Images annotated with landmarks around the nose and mouth	A pre-trained convolutional neural network (AlexNet) was trained to extract features from facial images. These were used as inputs for an SVM classifier. Four models were tested to classify images as unilateral or bilateral cleft or normal.	The proposed model with augmented images using high level features extracted from the CNN that served as input for the SVM classifier showed the best validation accuracy (92.22%) and testing accuracy (84.12%).
12.	Wu et al. (37)	USA	Detect the midfacial plane in individuals with cleft lip	3.2–6.7 months	50 subjects	AI based software was used to detect landmarks	3D facial images	Five 3D images of each subject were used to detect a mid-facial plane using five methods–direct placement, manual landmark, mirror method, deformation method and the machine learning method. The planes were rated for accuracy by three cleft surgeons, two craniofacial pediatricians and one craniofacial morphology researcher.	The manual based methods were rated the best of all. Of the computer based methods, the deformation method received the best scores. The machine learning method performed slightly better than the mirror method.
**III.PRE-SURGICAL ORTHOPEDICS**									
13.	Schiebl et al. (38)	Germany	Use sequential NAM plates generated using an AI software to reduce the size of the alveolar cleft	Neonates	17 infants with BCLP for development and 6 sets for validation	Development of an algorithm for automated processing	Digitized maxillary impressions	An algorithm was designed to segment the defective structures from a scanned maxillary model and create a mesh with virtual bridging of the defect. NAM plates were generated from the mesh. The plates were reshaped and resized to fit the maxilla as it grows.	The algorithm generated plates in 16 cases for 16 weeks of treatment. They were anatomically correct with minor deviations in the structure. On validation, 5 of the 6 plates were made by the algorithm. On assessment by a healthcare professional, 3 out of the 5 were evaluated as useful.
**IV.SPEECH ASSESSMENT**									
14.	Mathad et al. (39)	India	Evaluate misarticulated stops in participants with cleft palate (CP)	6–12 years	61 (31 participants with repaired cleft palate and 30 without cleft palate)	SVM classifier	Vowel onset points	A method is proposed to detect Vowel onset point to segment consonant-vowel transitions and analyse misarticulated stops. These are analyzed to train a SVM classifier that identifies misarticulated stops.	Vowel onset point detection by the proposed method showed 90% accuracy for normal speech and 75% for misarticulated speech. The SVM classifier obtained an accuracy of 90.57% based on VOP detected by the proposed method. Manual detection of VOPs increased the accuracy to 91.92%
15.	Dubey et al. (40)	India	Detect hypernasality in individuals with CP	7–12 years	60 (30 individuals without CP and 30 with CP)	SVM	Normalized harmonic amplitude, Harmonics amplitude ratio and prominent harmonics frequency	Speech recordings from participants were analyzed based on baseline features and proposed features. SVMs were used to classify speech based on the proposed features, individually and in combination. The results obtained were compared to those obtained by baseline features.	The highest level of accuracy was seen when the three proposed features–normalized harmonic amplitude, harmonic amplitude ratio and prominent harmonics frequency were used in combination (up to 87.89%). This was better than the individual features and baseline features.
16.	Wang et al. (41)	China	Detect hypernasality in individuals with CP	5–12 years	144 (72 children in each group)	Long Short Term Memory–Deep Recurrent Neural Network (LSTM–DRNN)	Linear Prediction Coefficients (LPC), Vocal tract area, reflection coefficients of vocal tract, formants + bandwidths, Vowel Space Area, GD spectrum, Voice Low tone to High tone Ratio, Power Spectrum Density, LPC spectrum, Long-term Spectral Flatness Measure, and Spectrum of Vocal Tract Impulse Response, Removing Glottal Excitation, Linear Prediction Coefficients Cepstrum (LPCC), and LPCC distance, Mel-Frequency Cepstral Coefficients (MFCCs), Mel spectrum, and 1/3 octave spectra	An automatic hypernasal speech detection system based on four category features–vocal shape based features, formant based features, vocal tract based cepstral features and vocal tract features. The results were compared to shallow classifiers using both mined features from the LSTM-DRNN and features without the mining process as input.	The LSTM–DRNN classifier had an accuracy of 92.67% when all four vocal tract based features were combined which was better than any shallow classifier. This was better than using the mined features as input for shallow classifiers as well as those not obtained by the mining process.
17.	Wutiwiwatchai (42)	Thailand	Detect nasal and oral phonemes in individuals with CP	7–12 years	60 (30 individuals with CP and 30 without CP)	Gausian Mixture Model (GMM) and DNN	Speech samples in the Thai language	A novel instrument was tested to asisst the speech therapist in assessment. DNN and GMM were used to identify oral and nasal phonemes correctly from mixed ora-nasal and oral only input of speech. The output sequence was compared with its correct phoneme sequence to compute the accuracy in identification.	The DNN showed better recognition than the GMM. Identification of phonemes was better in normal speech compared to that of cleft-palate. The oral-only input provided a better outcome than an oro-nasal input.
18.	Golabbakhsh et al. (43)	Iran	Detect hypernasality in participants with CLP	4–28 years	30 (15 individuals without CLP and 15 with CLP)	SVM	Jitter, shimmer, mel frequency cepstral coefficient (MFCC), bionic wavelet transform energy and bionic wavelet transform entropy	Recorded sentences from children were used to extract time and frequency features–jitter, shimmer, mel frequency cepstral coefficient (MFCC), bionic wavelet transform energy and bionic wavelet transform entropy. Using combinations of different features, hypernasal voice was classified from normal using SVM. The accuracy, sensitivity and specificity was evaluated against manual classification.	The use of mel frequency cepstral coefficient with bionic wavelet transform energy showed the highest accuracy and sensitivity though the values varied for every sentence.
19.	Liu et al. (44)	China	Classify the level of hypernasality in individuals with CP		64 (48 individuals of unrepaired CP and 16 without CP)	Back propagation neural network	Homomorphic spectrum sequence	Speech data containing four basic vowels is classified into levels of hypernasality. The feature extracted is the homomorphic Spectrum feature and a back propagation neural network is applied to classify the speech	The classification accuracy for different levels of hypernasality reached up to 80.7% and the accuracy for detection of normal or hypernasal speech reached 94.5%
20.	He et al. (45)	China, Australia	Detect resonance and consonant articulation disorders in individuals with CP	5–12 years	120 individuals with CP	GMM classifier	Short-time Shannon energy, non-linear Teager energy operator, mel frequency cepstral coefficient	Four acoustic features were extracted from the data and a GMM is used to classify speech based on individual features. Identification of consonant omission and consonant replacement was also done.	The system had the highest accuracy for classification of hypernasality based on the MFCC features (80.40%). Evaluation of consonant misarticulations had different accuracy for each consonant.
21.	He et al. (46)	China	Classify speech hypernasality and consonant articulation in individuals with of CP	5–12 years	120 individuals with CP	GMM classifier	Teager energy operator, first formant, number of formants	Participant recordings were classified as hypernasal or normal based on energy distribution ratio. A GMM classifier further detected one of the three levels of hypernasal speech using acoustic features. A method to detect initial consonant omission was also proposed based on the diiference in energy amplified frequency bands.	The accuracy of the system to detect levels of hypernasality is 80.74%. The classification accuracy for detecting consonant omission is 94.87%.
22.	Bocklet et al. (47)	Germany	Use phonemes to detect intelligibility in participants with cleft lip and palate (CLP)	Avg 7 years (1–17 years)	630 (380 individuals without CLP, 250 individuals with CLP)	SVM	Mel frequency cepstral coefficient, transformation matrices of Maximum Likelihood Linear Regression	Two approaches–a GMM and a Maximum Likelihood Linear Regression were used to model the articulatory space of a speaker based on speech data annotated by therapists. A Support Vector regression model was used to predict the level of intelligibility of the speech of CLP children based on vectors identified by the two approaches. Speech therapist labels were considered ground truth scores.	The maximum likelihood linear regression model was more effective in automatic phoneme evaluation which was in the same range as perceptual labels by speech therapists.
23.	He et al. (48)	China	Evaluate intelligibility and hypernasality in individuals with CP		240 words (uttered by individuals with CP)	GMM classifier	Shannon Energy, mel frequency cepstral coefficient	Two acoustic features were extracted. These were used to classify hypernasality and to detect speech intelligibility using GMM. The data was split into training and testing sets (80%-20%). Speech intelligibility was assessed by word recognition.	A combination of the two acoustic features was a better classifier for hypernasality than the features alone. The accuracy for hypernasality detection was up to 85.42%. The classification accuracy for speech intelligibility was 75% for normal but dropped with increase in levels to 16.5%.
**V.SURGERY**									
24.	Lin et al. (49)	Korea	Predict the need for orthognathic surgery using radiographs taken in childhood	5–7 years	56 cephalograms of individuals with UCLP	Random Forest and Xboost Algorithm	Cephalometric variables measured by an operator	Cephalometric parameters of participants with UCLP were assessed at the age of 5–7 years and later at 15 years. The data was processed by the Boruta method to determine cephalometric predictors for orthognathic surgery at an earlier age.	Significant cephalometric differences were found between participants needing surgery and those who did not. Four predictors–inclination of palatal plane to FH, ANB angle, combination factor and FCA were selected as predictors for need of orthognathic surgery in the future. The model had an accuracy of 87.4%, sensitivity of 97.83% and specificity of 90%.
25.	Li et al. (50)	China	Assist in placement of surgical markers in individuals	Neonates	2,568 images	DNN	Frontal facial images annotated with surgical markers	A dataset of pictures annotated with surgical markers was created. This was divided into training, validation and test set of pictures. After training the model, the test pictures were used to identify markers using the proposed model and compared to other methods of feature extraction.	The proposed model showed a better localization of surgical markers than other models used for image feature extraction.
26.	Park et al. (51)	Korea	Predict the need for orthognathic surgery using radiographs taken in childhood	9.3 years	131 Korean males (CLA: *n* = 35), UCLP: *n* = 56), BCLP: *n* = 40).	SVM	Cephalometric variables measured by an operator	A prediction model was made using forest wrapping that was based on cephalometric parameters. Accuracy was verified by a 10-fold cross validation test.	A total of 10 cephalometric variables were selected as predictors with an accuracy of 77.3%. The sensitivity of the model was 99% and specificity was 74.1%.

### Risk of Development of Cleft Lip and Palate

A total of six studies used AI to assess the risk of developing cleft lip and palate. They used family history (26), genetic information (27, 28, 30, 31), and structural data from the ToxRef toxicology database (29).

Two studies evaluated Single Nucleotide Polymorphisms (SNPs) for diagnostic and predictive value in individuals of non-syndromic cleft lip with or without palate (NSCL ± P). The study by Zhang et al. (30) validates the diagnostic ability of SNPs in the Han and Uyghur populations. They found that variations in two genes—methylenetetrahydrofolate reductase (MTHFR) and retinol-binding protein 4 (RBP4) play an essential role in the development of CL±P. Machado et al. (27) studied the Brazilian population for NSCL ± P and found interactions among 13 SNPs responsible and an important role played by genes involved in folate metabolism.

Two studies evaluated genotype interactions that contribute to the development of NSCL ± P. Li et al. (31) used two machine learning methods in a complementary manner to first identify SNPs associated with NSCL ± P in the Asian and European populations. They found strong evidence of interaction between SNPs in the WNT5B gene and the MAFB gene inducing NSCL ± P in both populations. Liu et al. (28) studied interactions among genes involved in cell adhesion. They identified multiway interactions among SNPs in 3 genes using machine learning algorithms.

Shafi et al. (26) tested the ability of multiple AI methods to predict the occurrence of cleft lip and palate based on the parents' history. They tested various AI methods to generate an accurate prediction for the occurrence of cleft lip and palate. Baker and colleagues (29) used AI to find chemicals associated with the development of the cleft palate and elaborated on the chain of events leading to this outcome.

### Diagnosis of Cleft Lip and Palate

Six studies used AI to identify clefts and morphologic features in individuals with cleft lip and palate. Studies detected clefts in a fetus (32) and using frontal photographs of infants (36). Jurek et al. (32) used AI to classify the ultrasound image of a fetus as normal or pathologic based on the presence of a cleft. Agarwal et al. (36) used AI to identify clefts using digital camera images. They used features from a previously trained convolutional neural network (CNN) for a support vector machine (SVM). This combination was able to identify images of individuals with a cleft from normal and could also classify unilateral and bilateral clefts. Zhang et al. estimated the cleft volume in children before secondary alveolar bone grafting using a neural network (33). They used a volumetric registration-based framework to reconstruct the defect and estimate its volume.

AI-based software was used in two studies to compare radiographic characteristics of individuals with clefts to individuals without clefts (34, 35). They demonstrated that participants with non-syndromic cleft lip and palate have skeletal and dental characteristics different from the normal controls. One study compared machine learning, manual, and other computer-based methods for detecting the mid-facial plane in individuals with unilateral and bilateral cleft lip and palate (37). The machine learning method used here was less accurate than the manual method, which was the most accurate among all three.

### Pre-surgical Orthopedics

One study used AI to develop sequential plates for infants with bilateral cleft lip and palate who required naso-alveolar molding (NAM) for reducing the severity of the cleft defect (38). Fabrication of successive plates is done by segmenting the structure, virtually bridging the gap, and generating plates. Molding of fetal tissues was done in subsequent stages, taking into account the growth of the maxillary arch using AI. A sequence of plates for NAM was thus generated, which could be 3D printed and delivered.

### Speech Assessment

Ten studies evaluated speech recognition in participants who had cleft lip and palate. The maximum use of AI was to study hypernasality. Three studies tried to detect its presence (40, 41, 43) and two classified it according to severity (44, 48). They used extracted features of speech as inputs for the classifiers. The identification of the severity of hypernasality was less accurate than detecting its presence. Studies also focused on phoneme detection (42) and their analysis (47) to assess the quality of speech. Evaluation of misarticulated stops (39), intelligibility (48) and resonance (45) was done using different models. Two studies detected consonant misarticulation (45, 46) in participants with cleft lip and palate. It was found that AI could detect a misarticulation but the accuracy varied for different consonants.

### Surgery

Three studies used AI as an aid in surgery. One study used annotated frontal facial photographs to identify surgical markers in infants with cleft lip and palate for surgical correction of the cleft lip (50). The objective was to reduce the variation in outcome due to the specialist's experience. Two studies tried to predict the need for orthognathic surgery based on cephalometric parameters at an early age (49, 51). They used radiographs taken at a young age (6 or 9 years) and compared these to those obtained at a later stage (15 years). AI models were used to detect features in the early radiographs that could later indicate the need for surgery.

## Discussion

Since Alan Turing asked the question, “Can machines think?” (52), humanity has been trying to make it a fact. From the first AI program, the Logic theorist (53), to the recently developed game of AlphaGo by Google (54), artificial intelligence has evolved to impact every aspect of human life. Despite being in a hiatus for years after conception, increased processing power and digital data have accelerated its development and application. Medical science is no exception. AI can aid in hospital administration, patient communication, data collection, diagnoses, outcome prediction, and help practitioners make decisions. The technology can significantly reduce the burden of disease on medical professionals and the burden of care for individuals and their guardians. We conducted a scoping review to find the current applications of AI in the care of cleft lip and/or palate (7). We found a total of 26 studies fulfilled the inclusion criteria. These studies were divided into five major categories based on the application of AI for individuals with cleft lip and/or palate. We did not apply any time restrictions to our search; nevertheless, the studies focusing on AI applications in cleft care were published during the last 10 years.

**Figure 2:**
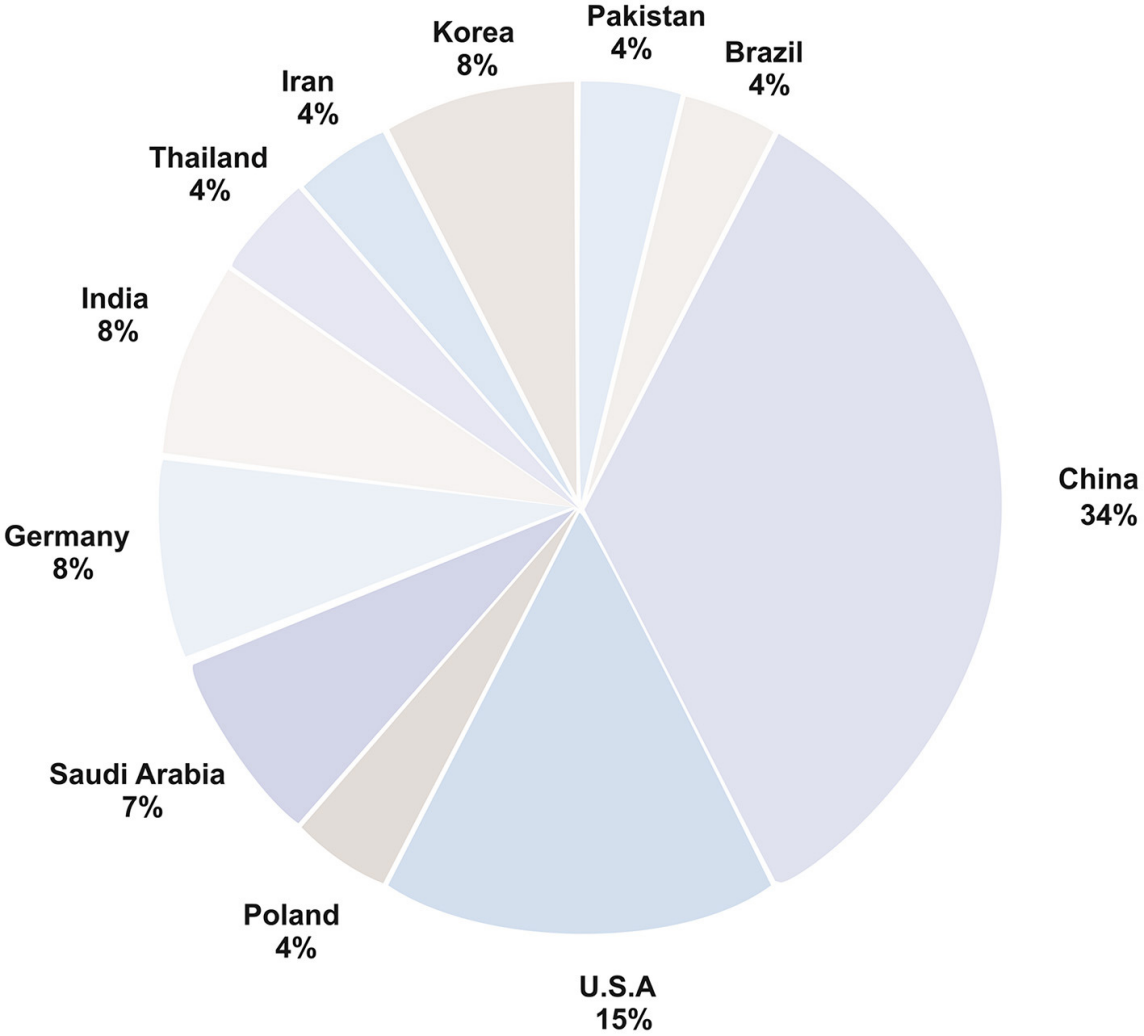
Country-wise distribution of AI based studies for individuals with cleft lip and palate.

Most of the studies in this review were carried out in China, with at least one study in each of the five areas originating from the country. The distribution of studies conducted in different countries is depicted in Figure 2. Though cleft lip and/or palate is a common birth defect, its prevalence varies among ethnicities (55). The high incidence of clefts in the Asian population compared with other ethnicities provides two possible reasons for the extensive research in China—to address a highly prevalent condition and availability of data. Most included studies used a relatively small dataset confined to the local population.

Studies that focused on detecting the risk of atypical development of non-syndromic cleft lip and palate will help contribute to a better understanding of this phenomenon. They can help discover environmental and genetic risk factors that prevent normal development and their mode of action. Such a study was carried out using known teratogens of cleft palate from the ToxRef database that identified the networked pathways leading to the cleft formation (29). They combined chemical structural data, U.S. Environment Protection Agency's high thoroughput screening data, and the published literature to propose the biologic pathways and develop a chemical-biologic predictive signature for cleft palate. Another study aimed to identify the risk of development using subjective answers to questionnaires (26). While the study developed a model with 92.6% accuracy on unseen data, the parameters reported significantly different between groups included factors such as smoking, consanguineous marriages, and medications that may vary widely across ethnicities and geographies. Studies that identified a genetic risk were based on detecting single nucleotide polymorphisms. These, too, were confined to a dataset obtained from specific populations such as the Brazilian (27) or the Asian population (30, 31). It is not possible to predict the accuracy, sensitivity, and specificity of these models when used in a different population. A means to expand the utility of these models is that the algorithms be made public so that other research groups can use diverse datasets to test and improve them.

Diagnosis of cleft lip and palate in the prenatal stages can help educate the caretakers of affected individuals with respect to their expectations and the needs of their ward. Diagnosing a cleft lip on ultrasound is readily done, but the same cannot be said for the diagnosis of cleft palate. The postnatal requirements for both conditions are vastly different. Cleft lip can be repaired surgically but the laterality of the cleft and its amplitude can influence the number of lip surgeries. On the other hand, the repair of a cleft palate may require multidisciplinary therapies over an extended period of time depending on the phenotype. The detection of prenatal clefts using 3D or 4D ultrasonography is possible but is not used widely as it is technically challenging and requires a steep learning curve for physicians (56). One study used pattern recognition to identify cleft palates on ultrasound images of fetuses in the first trimester by detecting hypoechogenic areas (32). Photographic assessments used standardized photographs for detection (36). Despite a high accuracy on the given dataset, the model may have poor clinical application when standardized, high-quality images are not available. Diagnosis of clefts using photographs will help provide education and facilitate affected individuals in seeking appropriate care.

Pre-surgical orthopedics aids in cleft repair by reducing its severity in infants with an alveolar defect. Nasoalveolar molding (NAM) is a therapy that provides a series of plates for the maxillary arch of the affected infant. These plates are sequentially modified to reduce the cleft and promote normal development of the arch. The traditional method of fabricating NAM plates involves making a series of impressions which are modified manually every week to adapt the plates to the changing anatomy of the arch. AI was used in one study (38) for the automatic generation of the sequence of these plates with modifications made virtually to the digital impressions. This approach will improve the quality of treatment and also save time by reducing the frequency of visits to the hospital. The model in this study was able to generate NAM plates for five out of the six test cases but these needed revisions. However, the algorithm could theoretically achieve integration of the pre-maxilla and prevent the collapse of the segments.

Speech assessment was based on extracted features and had maximum studies. These provide encouraging experimental results, but they do not reflect the practical performance of these systems. They characterize speech based on hypernasality (40, 41, 43, 44, 46, 48), identifying phonemes (42, 47) and misarticulations (39, 45). These studies use features of speech data at a single point in time. The applicability of these systems to different clinical scenarios—where a complete word or sentence is likely to be used as input or the individual's voice will change with age—needs to be investigated. Further, these systems have been developed, trained, and tested using regional languages and populations. The generalization of the same to populations that speak a different tongue and have diverse features of speech requires testing.

Surgical repair is an important clinical intervention to correct orofacial cleft. The procedure is carried out by highly trained specialists who may not be available in rural hospital settings. To overcome this limitation, one study trained an AI model to identify surgical markers on photographs of infants who need cleft lip repair (50). Another useful application for AI was predicting the need for orthognathic surgery at an early age (49, 51). These models were used to identify cephalometric parameters that can indicate the future need for orthognathic surgery. However, their performance on external datasets is yet to be established.

Data can be a scarce resource in the medical sciences depending on the prevalence of craniofacial abnormality and ethical concerns. To overcome this limitation, studies successfully used augmentation techniques to create datasets that provide accurate results (36). A clinic, however, is not likely to see only those individuals who are normal or have clefts. Individuals with different facial types and other facial deformities will also present for treatment. Creating a variation in the dataset accordingly is necessary to train the model. Validating the model using an independent data set and not a subset of data collected during development will help identify the actual performance of the model.

The sole availability of data can help develop and train an AI model, as seen with accuracy above 94% in some models in this review. The specificity and sensitivity of the models were also above 90% in a few cases. Their testing, however, has been done in a controlled environment. It does not mean that the model will have a similar performance in a real-life clinical scenario, where the data available is different in quality and quantity. Studies used pre-determined datasets with known cases of clefts in a very high proportion. This does not align with the prevalence of the condition, which is known to be far less (8–10). Over-representation of a condition using enriched datasets can lead to bias in the system with limited applicability in an external setting. An AI program needs large amounts of data, but such representation can only aid in developing an experimental model. Including consecutive participants from clinics in the training and testing data will help the developed models perform better in a hospital setting.

Few studies used clinical data to produce predictive models that can assist clinicians directly. Studies that focused on developing AI models for predicting the occurrence of cleft lip and palate using patient history (26), detecting surgical markers (50), and predicting surgery at later stages (49, 51) are among them. The study that used demographic data to predict clefts achieved an accuracy of 92.6%. It included various cultural influences in the input data, which may not be prevalent in other populations. The model could thus be less accurate in the general population. Reproducing the study and adding more demographic data can help strengthen this AI-based approach. Another way forward can be combining the data with genetic information such as that used by other AI models (27, 28, 30) and better predicting the likelihood of occurrence. Training a model on more information, in both quantity and variation, will help achieve more accurate predictions. They can then be used to direct clinicians on providing prenatal counseling, using preventive approaches, and preparing the parents' expectations for the future. These predictive algorithms, along with another, such as one that can determine pathways of development of clefts via toxic exposure (29), can be used as public health tools to prevent the development of clefts or create awareness in a local population when prevention is not possible.

The presented scoping review has certain limitations. Though we tried to be thorough in our search by using artificial intelligence as a MeSH term, it is possible that specific keywords from the field of machine learning such as neural networks, support vector machines, and supervised or unsupervised learning could produce more results. Researchers who undertake similar projects in this area can focus upon these specific fields to gain maximum results.

## Conclusion

AI appears to be an optimistic approach that can help individuals with cleft lip and/or palate. Currently, it has been used for predicting the risk of development of non-syndromic cleft lip and palate, for diagnosis—prenatal, photographic, and identification of cephalometric characteristics and mid-facial plane in participants. AI has also been used to aid in pre-surgical orthopedics, detect speech pathology, and predict the need for surgery. The models developed so far show promising results in controlled settings. However, they have not been prospectively tested in different clinical settings, and thus, their applicability cannot be generalized. Further research in this area with the focus on developing patient-centric AI models for prenatal diagnosis, postnatal diagnosis to identify the treatment needs based on the extent of the cleft defect and for the assistance in the treatment. Future studies with multi-centric approach with cross country and cross center validation of AI-based models are highly recommended for generalizing such models for widespread clinical applications.

## Data Availability Statement

The raw data supporting the conclusions of this article will be made available by the authors, without undue reservation.

## Author Contributions

HD and PC conceived and designed the study. HD, PC, R-FK, RS, and KD wrote the manuscript and analyzed the results. R-FK, MA, and SA provided critical reading and comments. All authors read and approved the final draft of the manuscript.

## Conflict of Interest

The authors declare that the research was conducted in the absence of any commercial or financial relationships that could be construed as a potential conflict of interest.

## Publisher's Note

All claims expressed in this article are solely those of the authors and do not necessarily represent those of their affiliated organizations, or those of the publisher, the editors and the reviewers. Any product that may be evaluated in this article, or claim that may be made by its manufacturer, is not guaranteed or endorsed by the publisher.

## References

[1] Steimann F. Fuzzy set theory in medicine. Artif Intell Med. (1997) 11:1–7. 9267588 10.1016/s0933-3657(97)00019-5

[2] Shortliffe EH Davis R Axline SG Buchanan BG Green CC Cohen SN. Computer-based consultations in clinical therapeutics: explanation and rule acquisition capabilities of the MYCIN system. Comput Biomed Res. (1975) 8:303–20. 10.1016/0010-4809(75)90009-9 1157471

[3] Jiang F Jiang Y Zhi H Dong Y Li H Ma S. Artificial intelligence in healthcare: past, present and future. Stroke Vasc Neurol. (2017) 2:230–43. 10.1136/svn-2017-000101 29507784 PMC5829945

[4] Shan T Tay FR Gu L. Application of artificial intelligence in dentistry. J Dent Res. (2021) 100:232–44. 10.1177/0022034520969115 33118431

[5] Kulkarni S Seneviratne N Baig MS Khan AHA. Artificial intelligence in medicine: where are we now? Acad Radiol. (2020) 27:62–70. 10.1016/j.acra.2019.10.001 31636002

[6] Prevalence of Cleft Lip & Cleft Palate. National Institute of Dental and Craniofacial Research. Available online at: https://www.nidcr.nih.gov/research/data-statistics/craniofacial-birth-defects/prevalence (accessed December 15, 2020).

[7] Tolarova MM. Global health issues related to cleft lip and palate: prevention and treatment need to team together. Indian J Dent Res. (2016) 27:455. 10.4103/0970-9290.195607 27966497

[8] Cobourne MT. The complex genetics of cleft lip and palate. Eur J Orthod. (2004) 26:7–16. 10.1093/ejo/26.1.7 14994877

[9] Yilmaz HN Özbilen EÖ Üstün T. The prevalence of cleft lip and palate patients: a single-center experience for 17 Years. Turk J Orthod. (2019) 32:139–44. 10.5152/TurkJOrthod.2019.18094 31565688 PMC6756567

[10] Vyas T Gupta P Kumar S Gupta R Gupta T Singh HP. Cleft of lip and palate: a review. J Fam Med Primary Care. (2020) 9:2621. 10.4103/jfmpc.jfmpc_472_20 PMC7491837 32984097

[11] Toobaie A Yousef Y Balvardi S St-Louis E Baird R Guadagno E. Incidence and prevalence of congenital anomalies in low- and middle-income countries: a systematic review. J Pediatr Surg. (2019) 54:1089–93. 10.1016/j.jpedsurg.2019.01.034 30786990

[12] Stone C. Cleft lip and palate: etiology, epidemiology, preventive and intervention strategies. Anat Physiol. (2013) 4:1–6. 10.4172/2161-0940.1000150

[13] Chaudhari PK Kharbanda OP Chaudhry R Pandey RM Chauhan S Bansal K. Factors affecting high caries risk in children with and without cleft lip and/or palate: a cross-sectional study. Cleft Palate Craniofac J. (2020). 10.1177/1055665620980206. [Epub ahead of print]. 33349037

[14] Poenaru D Lin D Corlew S. Economic valuation of the global burden of cleft disease averted by a large cleft charity. World J Surg. (2016) 40:1053–9. 10.1007/s00268-015-3367-z 26669788

[15] Mednick L Snyder J Schook C Blood EA Brown S-E Weatherley-White RCA. Causal attributions of cleft lip and palate across cultures. Cleft Palate Craniofac J. (2013) 50:655–61. 10.1597/11-300R1 23030676

[16] Wehby GL Cassell CH. The impact of orofacial clefts on quality of life and healthcare use and costs. Oral Dis. (2010) 16:3–10. 10.1111/j.1601-0825.2009.01588.x 19656316 PMC2905869

[17] Rau A Ritschl LM Mücke T Wolff K-D Loeffelbein DJ. Nasoalveolar molding in cleft care–experience in 40 patients from a single centre in Germany. PLoS ONE. (2015) 10:e0118103. 10.1371/journal.pone.0118103 25734535 PMC4347986

[18] Johansson B Ringsberg KC. Parents' experiences of having a child with cleft lip and palate. J Adv Nurs. (2004) 47:165–73. 10.1111/j.1365-2648.2004.03075.x 15196190

[19] Chaudhari PK Dhingra K Zere E. Digital presurgical infant orthopedics in COVID-19 crisis. Cleft Palate Craniofac J. (2020). 10.1177/1055665620980230. [Epub ahead of print]. 33327776

[20] Shanbhag G Pandey S Mehta N Kini Y Kini A. A virtual noninvasive way of constructing a nasoalveolar molding plate for cleft babies, using intraoral scanners, CAD, and prosthetic milling. Cleft Palate Craniofac J. (2020) 57:263–6. 10.1177/1055665619886476 31698948

[21] Batra P Gribel BF Abhinav BA Arora A Raghavan S. OrthoAligner “NAM”: a case series of presurgical infant orthopedics (PSIO) using clear aligners. Cleft Palate Craniofac J. (2020) 57:646–55. 10.1177/1055665619889807 31795731

[22] Schütte S Acevedo PNM Flahault A. Health systems around the world - a comparison of existing health system rankings. J Glob Health. (2018) 8:010407. 10.7189/jogh.08-010407 29564084 PMC5857204

[23] Munn Z Peters MDJ Stern C Tufanaru C McArthur A Aromataris E. Systematic review or scoping review? Guidance for authors when choosing between a systematic or scoping review approach. BMC Med Res Methodol. (2018) 18:143. 10.1186/s12874-018-0611-x 30453902 PMC6245623

[24] Peters MDJ Godfrey C McInerney P Munn Z Tricco AC Khalil H. Chapter 11: Scoping Reviews (2020 version). In: Aromataris E, Munn Z, editors. JBI Manual for Evidence Synthesis, Joanna Briggs Institute; The University of Adelaide (2020). Available online at: https://synthesismanual.jbi.global

[25] Tricco AC Lillie E Zarin W O'Brien KK Colquhoun H Levac D. PRISMA extension for scoping reviews (PRISMA-ScR): checklist and explanation. Ann Intern Med. (2018) 169:467–73. 10.7326/M18-0850 30178033

[26] Shafi N Bukhari F Iqbal W Almustafa KM Asif M Nawaz Z. Cleft prediction before birth using deep neural network. Health Informatics J. (2020) 26:2568–85. 10.1177/1460458220911789 32283987

[27] Machado RA de Oliveira Silva C Martelli-Junior H das Neves LT Coletta RD. Machine learning in prediction of genetic risk of nonsyndromic oral clefts in the Brazilian population. Clin Oral Invest. (2021) 25:1273–80. 10.1007/s00784-020-03433-y 32617779

[28] Liu D Wang M Yuan Y Schwender H Wang H Wang P. Gene–gene interaction among cell adhesion genes and risk of nonsyndromic cleft lip with or without cleft palate in Chinese case-parent trios. Mol Genet Genomic Med. (2019) 7:e00872. 10.1002/mgg3.872 31419083 PMC6785639

[29] Baker NC Sipes NS Franzosa J Belair DG Abbott BD Judson RS. Characterizing cleft palate toxicants using ToxCast data, chemical structure, and the biomedical literature. Birth Defects Rese. (2020) 112:19–39. 10.1002/bdr2.1581 31471948 PMC8454266

[30] Zhang S-J Meng P Zhang J Jia P Lin J Wang X. Machine learning models for genetic risk assessment of infants with non-syndromic orofacial cleft. Genom Proteom Bioinformat. (2018) 16:354–64. 10.1016/j.gpb.2018.07.005 30578914 PMC6364041

[31] Li Q Kim Y Suktitipat B Hetmanski JB Marazita ML Duggal P. Gene-gene interaction among WNT genes for oral cleft in trios. Genet Epidemiol. (2015) 39:385–94. 10.1002/gepi.21888 25663376 PMC4469492

[32] Jurek J Wójtowicz W Wójtowicz A. Syntactic pattern recognition-based diagnostics of fetal palates. Pattern Recogn Lett. (2020) 133:144–50. 10.1016/j.patrec.2020.02.023

[33] Zhang Y Pei Y Chen S Guo Y Ma G Xu T. Volumetric registration-based cleft volume estimation of alveolar cleft grafting procedures. In: 2020 IEEE 17th International Symposium on Biomedical Imaging (ISBI). Iowa City, IA (2020). p. 99–103. 10.1109/ISBI45749.2020.9098407

[34] Alam MK Alfawzan AA. Dental characteristics of different types of cleft and non-cleft individuals. Front Cell Dev Biol. (2020) 8:789. 10.3389/fcell.2020.00789 32984313 PMC7477047

[35] Alam MK Alfawzan AA. Evaluation of sella turcica bridging and morphology in different types of cleft patients. Front Cell Dev Biol. (2020) 8:656. 10.3389/fcell.2020.00656 32793599 PMC7387404

[36] Agarwal S Hallac RR Mishra R Li C Daescu O Kane A. Image based detection of craniofacial abnormalities using feature extraction by classical convolutional neural network. In: 2018 IEEE 8th International Conference on Computational Advances in Bio and Medical Sciences (ICCABS). Las Vegas, NV (2018). p. 1–6. 10.1109/ICCABS.2018.8541948

[37] Wu J Heike C Birgfeld C Evans K Maga M Morrison C. Measuring symmetry in children with unrepaired cleft lip: defining a standard for the three-dimensional midfacial reference plane. Cleft Palate Craniofac J. (2016) 53:695–704. 10.1597/15-053 26752127

[38] Schiebl J Bauer FX Grill F Loeffelbein DJ. RapidNAM: algorithm for the semi-automated generation of nasoalveolar molding device designs for the presurgical treatment of bilateral cleft lip and palate. IEEE Trans Biomed Eng. (2020) 67:1263–71. 10.1109/TBME.2019.2934907 31403406

[39] Mathad VC Prasanna SM. Vowel onset point based screening of misarticulated stops in cleft lip and palate speech. IEEE/ACM Transac Audio Speech Language Process. (2019) 28:450–60. 10.1109/TASLP.2019.2957887

[40] Dubey AK Prasanna SRM Dandapat S. Sinusoidal model-based hypernasality detection in cleft palate speech using CVCV sequence. Speech Commun. (2020) 124:1–12. 10.1016/j.specom.2020.08.001

[41] Wang X Yang S Tang M Yin H Huang H He L. HypernasalityNet: deep recurrent neural network for automatic hypernasality detection. Int J Med Informat. (2019) 129:1–12. 10.1016/j.ijmedinf.2019.05.023 31445242

[42] Wutiwiwatchai C Chootrakool P Kasuriya S Makarabhirom K Ooppanasak N Prathanee B. Naso-articulometry speech database for cleft-palate speech assessment. In: 2018 Oriental COCOSDA - International Conference on Speech Database and Assessments. Miyazaki (2018). p. 32–6. 10.1109/ICSDA.2018.8693008

[43] Golabbakhsh M Abnavi F Kadkhodaei Elyaderani M Derakhshandeh F Khanlar F Rong P. Automatic identification of hypernasality in normal and cleft lip and palate patients with acoustic analysis of speech. J Acoust Soc Am. (2017) 141:929. 10.1121/1.4976056 28253654

[44] Liu Y Wang X Hang Y He L Yin H Liu C. Hypemasality detection in cleft palate speech based on natural computation. In: 2016 12th International Conference on Natural Computation, Fuzzy Systems and Knowledge Discovery (ICNC-FSKD). Changsha (2016). p. 523–8. 10.1109/FSKD.2016.7603228

[45] He L Tan J Hao H Tang M Yin H Lech M. Automatic evaluation of resonance and articulation disorders in cleft palate speech. In: 2015 IEEE China Summit and International Conference on Signal and Information Processing (ChinaSIP). IEEE (2015). p. 358–62. 10.1109/ChinaSIP.2015.7230424

[46] He L Zhang J Liu Q Yin H Lech M. Automatic evaluation of hypernasality and consonant misarticulation in cleft palate speech. IEEE Sign Process Lett. (2014) 21:1298–301. 10.1109/LSP.2014.2333061

[47] Bocklet T Riedhammer K Eysholdt U Nöth E. Automatic phoneme analysis in children with Cleft Lip and Palate. In: 2013 IEEE International Conference on Acoustics, Speech and Signal Processing. Vancouver, BC (2013) p. 7572–6. 10.1109/ICASSP.2013.6639135

[48] He L Zhang J Liu Q Yin H Lech M. Automatic evaluation of hypernasality and speech intelligibility for children with cleft palate. In: 2013 IEEE 8th Conference on Industrial Electronics and Applications (ICIEA). Melbourne: IEEE (2013). p. 220–3.

[49] Lin G Kim P-J Baek S-H Kim H-G Kim S-W Chung J-H. Early prediction of the need for orthognathic surgery in patients with repaired unilateral cleft lip and palate using machine learning and longitudinal lateral cephalometric analysis data. J Craniofac Surg. (2021) 32:616–20. 10.1097/SCS.0000000000006943 33704994

[50] Li Y Cheng J Mei H Ma H Chen Z Li Y. CLPNet: cleft lip and palate surgery support with deep learning. In: 2019 41st Annual International Conference of the IEEE Engineering in Medicine and Biology Society (EMBC). (2019). p. 3666–72. 10.1109/EMBC.2019.8857799 31946672

[51] Park H-M Kim P-J Kim H-G Kim S Baek S-H. Prediction of the need for orthognathic surgery in patients with cleft lip and/or palate. J Craniofac Surg. (2015) 26:1159–62. 10.1097/SCS.0000000000001605 26080148

[52] Turing A. Computing machinery and intelligence-AM Turing. Mind. (1950) 49:433–60. 10.1093/mind/LIX.236.433

[53] Gugerty L. Newell and Simon's logic theorist: historical background and impact on cognitive modeling. In: Proceedings of the Human Factors and Ergonomics Society Annual Meeting. Los Angeles, CA: SAGE Publications Sage CA (2006). p. 880–4. 10.1177/154193120605000904

[54] Holcomb SD Porter WK Ault SV Mao G Wang J. Overview on deepmind and its alphago zero AI. In: Proceedings of the 2018 International Conference on Big Data and Education. Honolulu, HI (2018). p. 67–71. 10.1145/3206157.3206174

[55] Mossey PA Shaw WC Munger RG Murray JC Murthy J Little J. Global oral health inequalities: challenges in the prevention and management of orofacial clefts and potential solutions. Adv Dent Res. (2011) 23:247–58. 10.1177/0022034511402083 21490237 PMC6699117

[56] James JN Schlieder DW. Prenatal counseling, ultrasound diagnosis, and the role of maternal-fetal medicine of the cleft lip and palate patient. Oral Maxillofac Surg Clin North Am. (2016) 28:145–51. 10.1016/j.coms.2015.12.005 26928557

